# Supplementary material for: DYRK1A signalling synchronizes the mitochondrial import pathways for metabolic rewiring
Source: Nat Commun. 2024 Jun 20;15:5265. doi: 10.1038/s41467-024-49611-4 (PMC11189921; doi:10.1038/s41467-024-49611-4)
Supplement: Supplementary file 1 — Supplementary Information [file 41467_2024_49611_MOESM1_ESM.pdf]

Supplementary Information

DYRK1A signalling synchronizes the mitochondrial import pathways for metabolic rewiring

Adinarayana Marada[1,10], Corvin Walter[1,2,10], Tamara Suhm[1], Sahana Shankar[1], Arpita Nandy[1,2,3], Tilman Brummer[4,5,6], Ines Dhaouadi[1], F.-Nora Vögtle[7,8,9,*] and Chris Meisinger[1,5,9,*]

[1]Institute of Biochemistry and Molecular Biology, ZBMZ, Faculty of Medicine, University of Freiburg, 79104 Freiburg, Germany.
[2]Faculty of Biology, University of Freiburg, 79104 Freiburg, Germany.
[3]Spemann Graduate School of Biology and Medicine, University of Freiburg, 79104 Freiburg, Germany.
[4]Institute of Molecular Medicine, ZBMZ, Faculty of Medicine, University of Freiburg, 79104 Freiburg, Germany.
[5]BIOSS Centre for Biological Signalling Studies, University of Freiburg, 79104 Freiburg, Germany.
[6]German Cancer Consortium DKTK Partner Site Freiburg, German Cancer Research Center (DKFZ), Heidelberg, Germany
[7]Center for Molecular Biology of Heidelberg University (ZMBH), DKFZ-ZMBH Alliance, 69120 Heidelberg, Germany
[8]Network Aging Research, Heidelberg University, 69120 Heidelberg, Germany,
[9]CIBSS - Centre for Integrative Biological Signalling Studies, University of Freiburg, 79104 Freiburg, Germany

[10]These authors contributed equally.

*Correspondence: chris.meisinger@biochemie.uni-freiburg.de or n.voegtle@zmbh.uni-heidelberg.de

Supplementary Information including:
Supplementary Figures 1 and 2

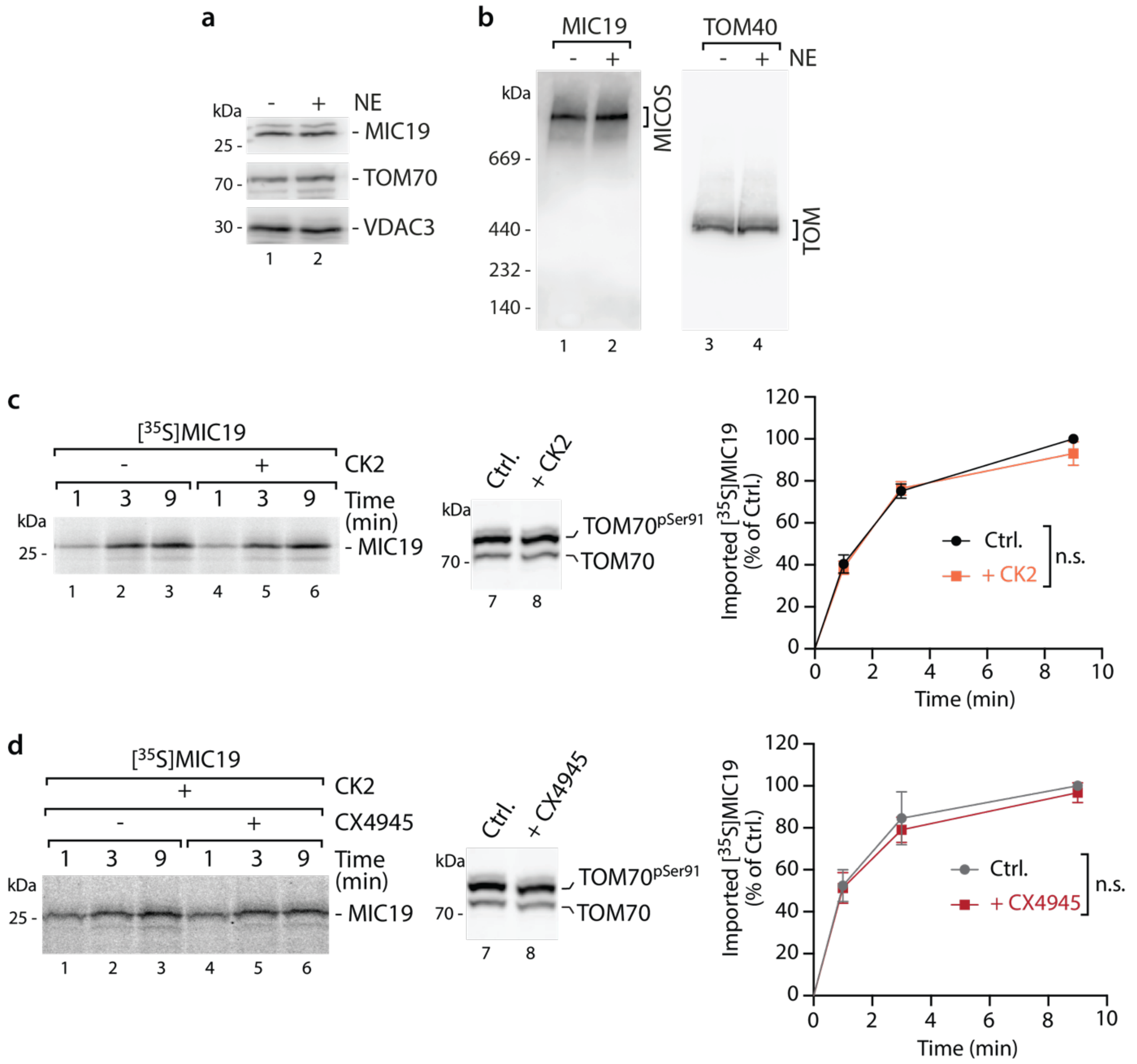

Supplementary Figure 1 | MIC19 import and TOM70 phosphorylation are independent of NE stimulation and Casein kinase 2.

a, Immunoblotting of isolated mitochondria from PIBA cells incubated in the presence or absence of NE for 18 h (see Figure 1b) after SDS-PAGE and analyzed with antisera against indicated proteins.

b, Immunoblotting of isolated mitochondria from PIBA cells incubated in the presence or absence of NE for 18 h (see Figure 1b) after BN-PAGE and analyzed with antisera against indicated proteins.

c, Import of [^{35}S]MIC19 precursor protein into mitochondria after incubation in the absence or presence of CK2. For quantification the control reaction (Ctrl., without CK2 pre-incubation) at 9 min import time point was set to 100%. Data represent mean ± SEM from three independent experiments. A multiple paired t test with a false discovery rate (FDR) of 1% and a two-stages step-up method of Benjamini, Krieger and Yekutieli was performed to compare between two groups. See the methods section for details on statistical analyses. The TOM70 phosphorylation status was monitored by Phos-tag gels (lanes 7 and 8). n.s., not significant.

d, Import of [^{35}S]MIC19 precursor protein in mitochondria treated with CK2 in the presence or absence of the CK2 inhibitor CX4945 (23). Experiment was performed as in **c**. Source data are provided as a Source Data file.

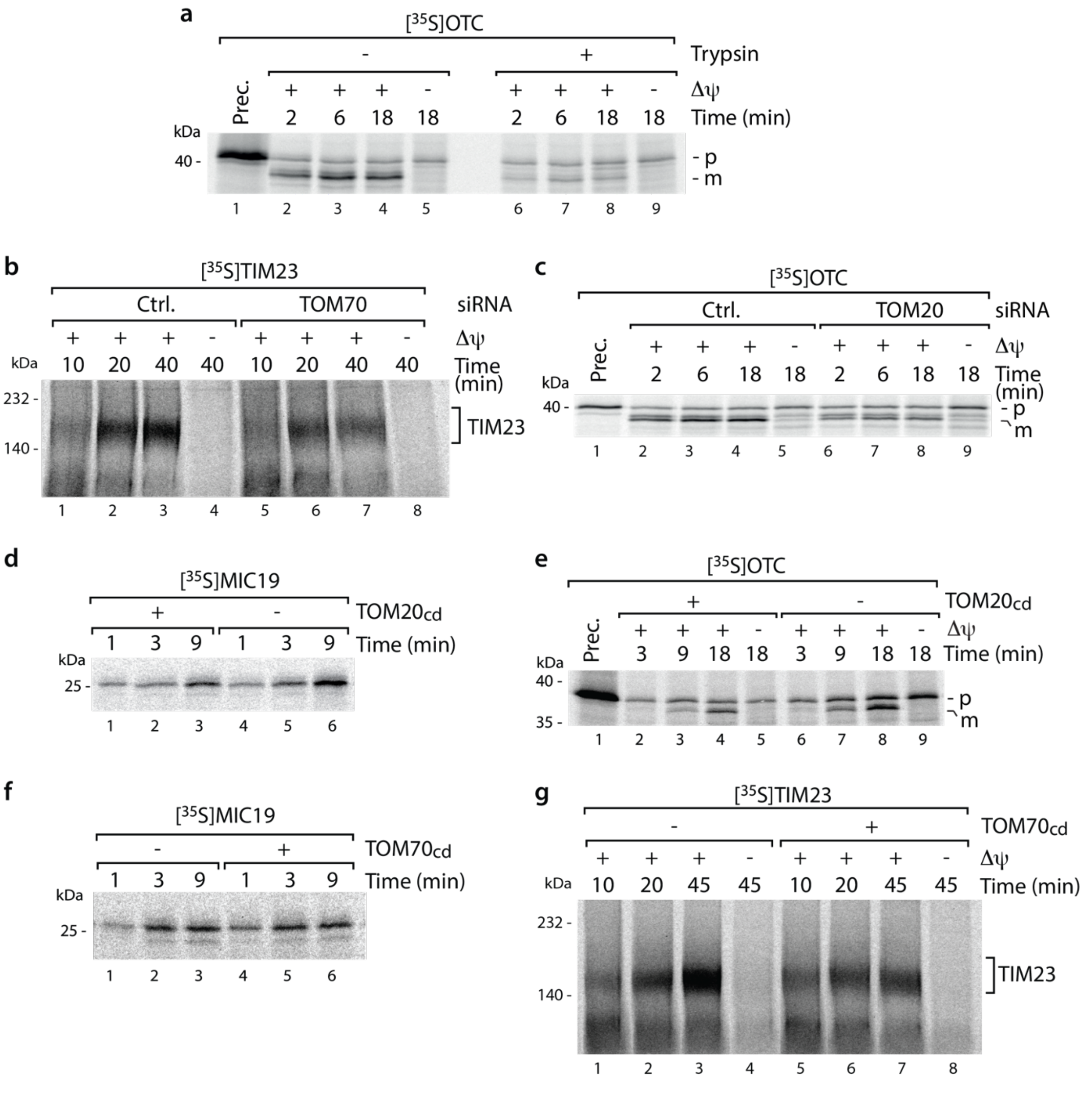

Supplementary Figure 2 | Human MIC19 import into mitochondria requires TOM20.

a. Import of [^{35}S]OTC precursor protein as model substrate of the presequence import pathway into mitochondria that were treated in the absence or presence of Trypsin. Non-imported precursor was removed by Proteinase K. Samples were analyzed by SDS-PAGE and imported OTC was detected by autoradiography.

b. Import of [^{35}S]TIM23 precursor protein as model substrate of the carrier import pathway in TOM70- and Mock (Ctrl.) depleted cells. Samples were lysed in digitonin buffer and subjected to BN-PAGE and autoradiography.

c. Import of [^{35}S]OTC precursor protein into mitochondria from TOM20- and mock (Ctrl.) depleted cells. Analysis as in **a.**

d. Import of [^{35}S]MIC19 precursor protein into mitochondria in the presence or absence of 1 µg purified TOM20 cytosolic domain (TOM20$_{cd}$). Samples were analyzed by SDS-PAGE and imported MIC19 was detected by autoradiography.

e. Import of [^{35}S]OTC precursor protein into mitochondria in the presence or absence of 1 µg purified $TOM20_{cd}$. Analysis as in **d.**
f. Import of [^{35}S]MIC19 precursor protein into mitochondria in the presence or absence of 1 µg purified $TOM70_{cd}$. Analysis as in **d.**
g. Import of [^{35}S]TIM23 precursor protein into mitochondria in the presence or absence of 1 µg purified $TOM70_{cd}$. Samples were lysed in digitonin buffer and subjected to BN-PAGE and autoradiography.
