# Supplementary material for: DYRK1A signalling synchronizes the mitochondrial import pathways for metabolic rewiring
Source: Nat Commun. 2024 Jun 20;15:5265. doi: 10.1038/s41467-024-49611-4 (PMC11189921; doi:10.1038/s41467-024-49611-4)

Figure 1b

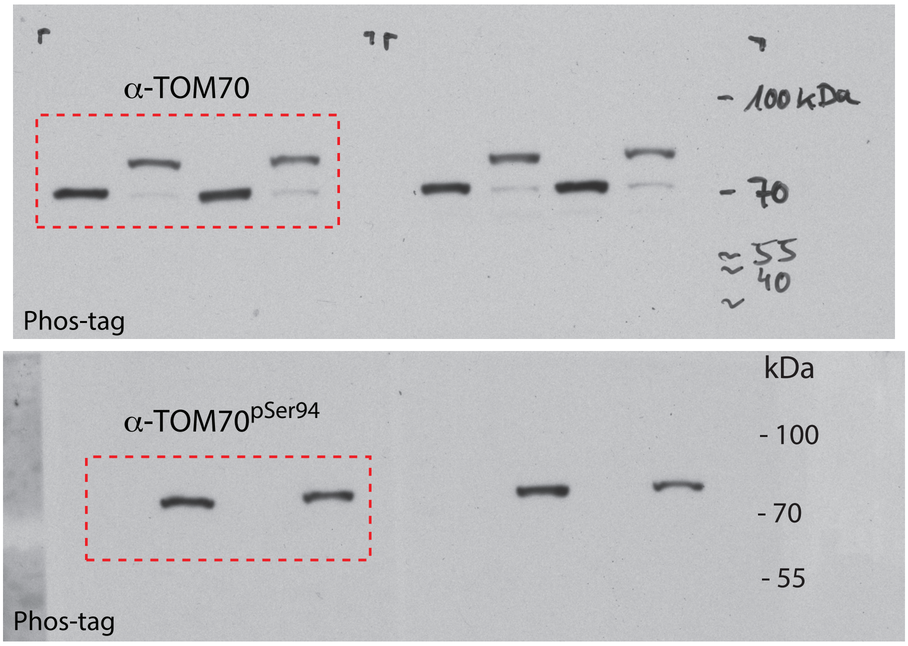

Figure 1d

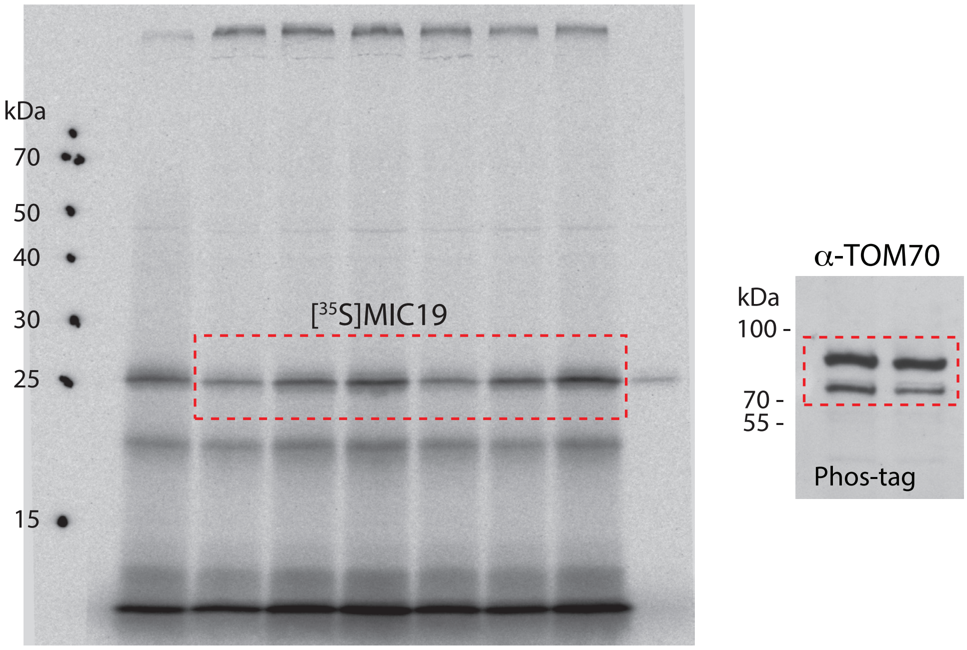

Figure 2c

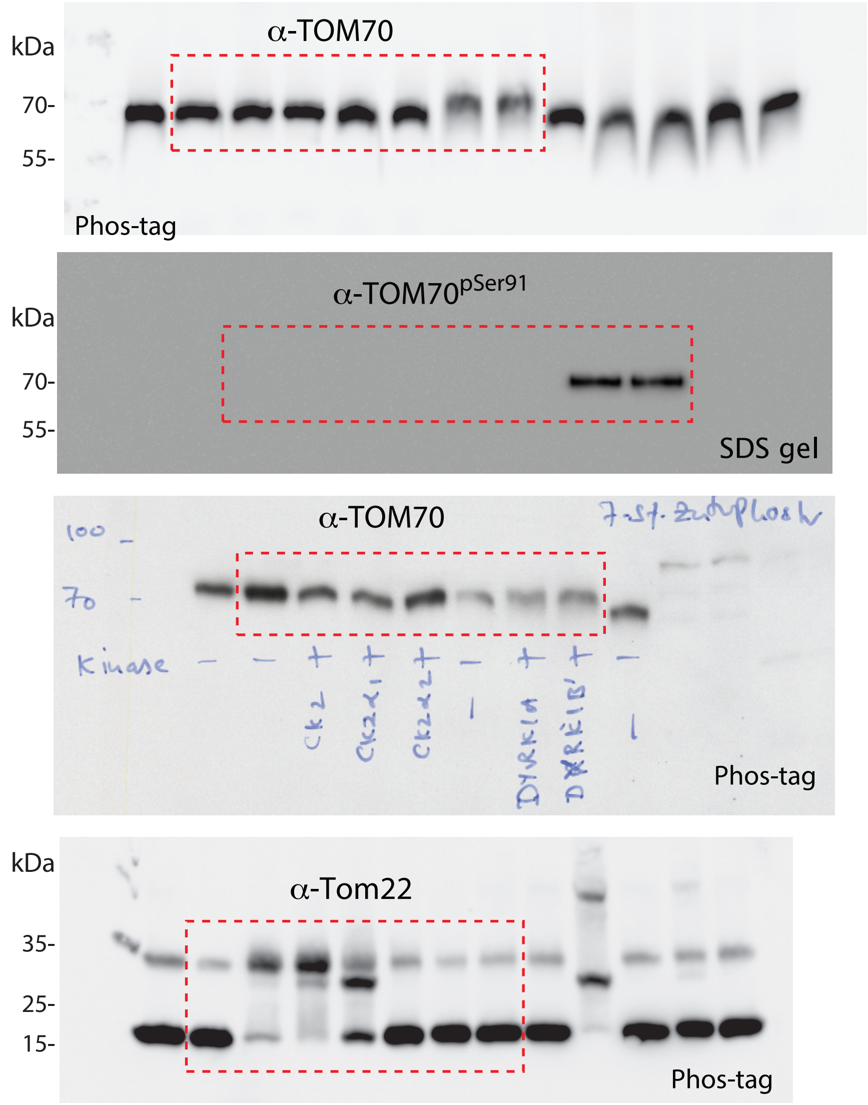

Figure 2d

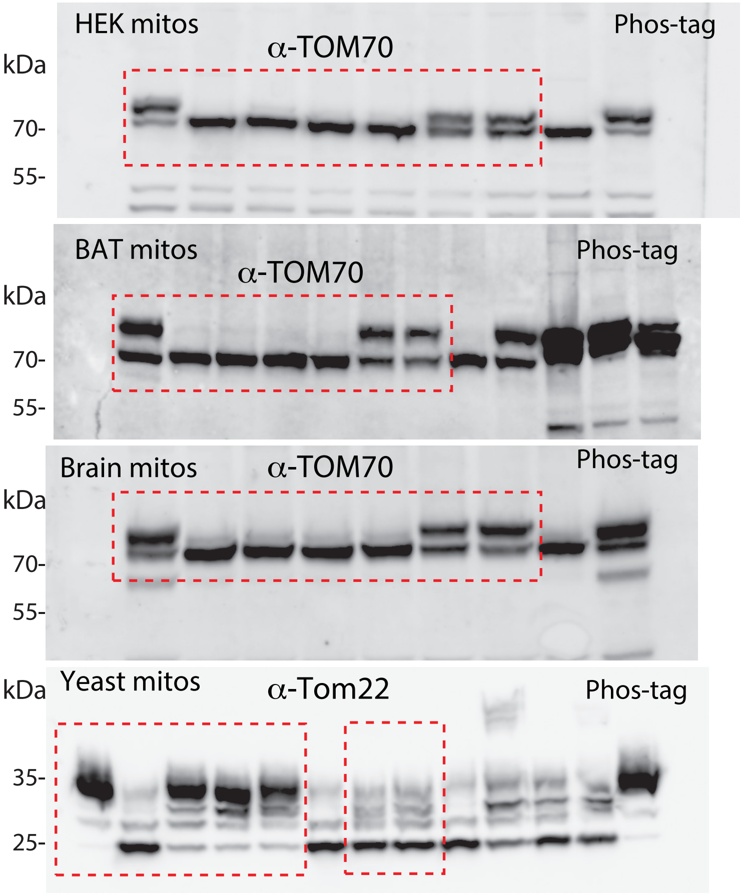

Figure 2e

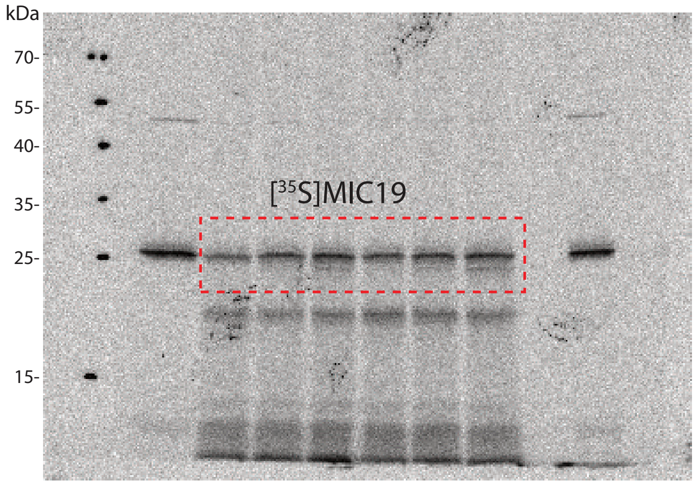

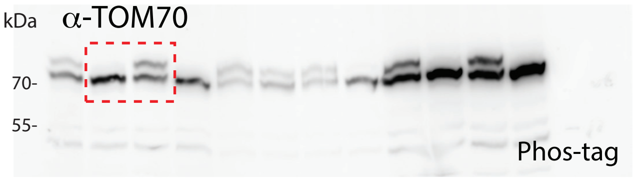

Figure 2f

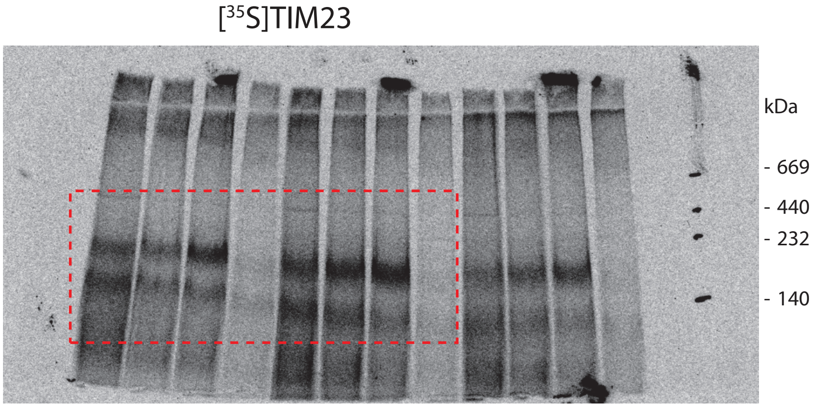

Figure 3a

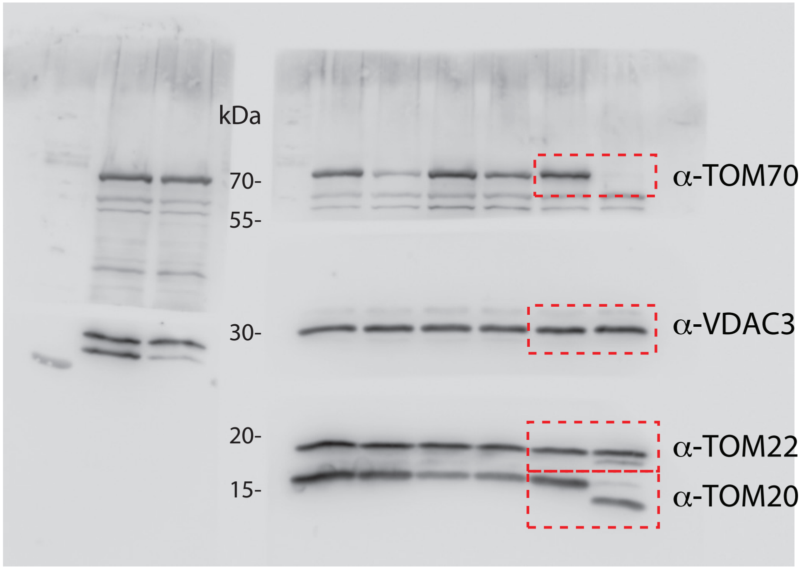

Figure 3b

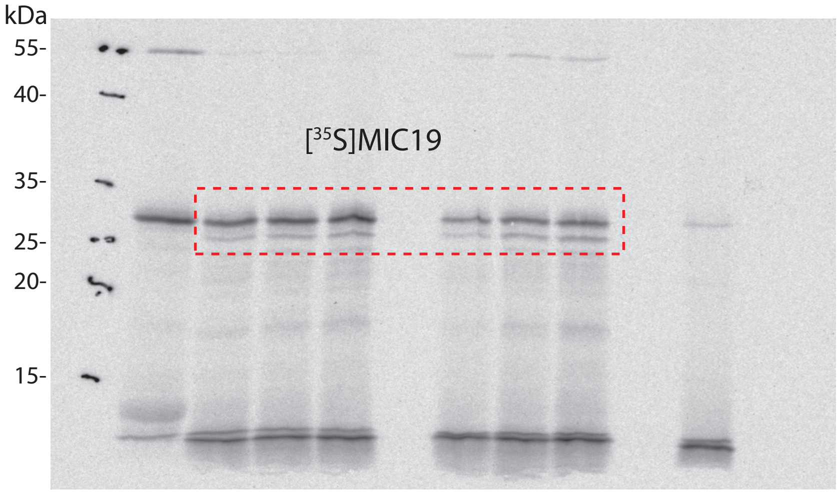

Figure 3c

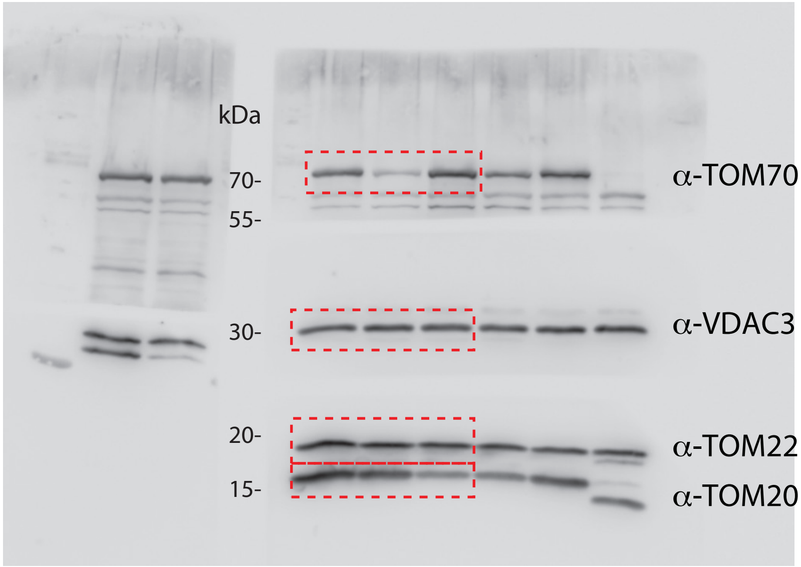

Figure 3e

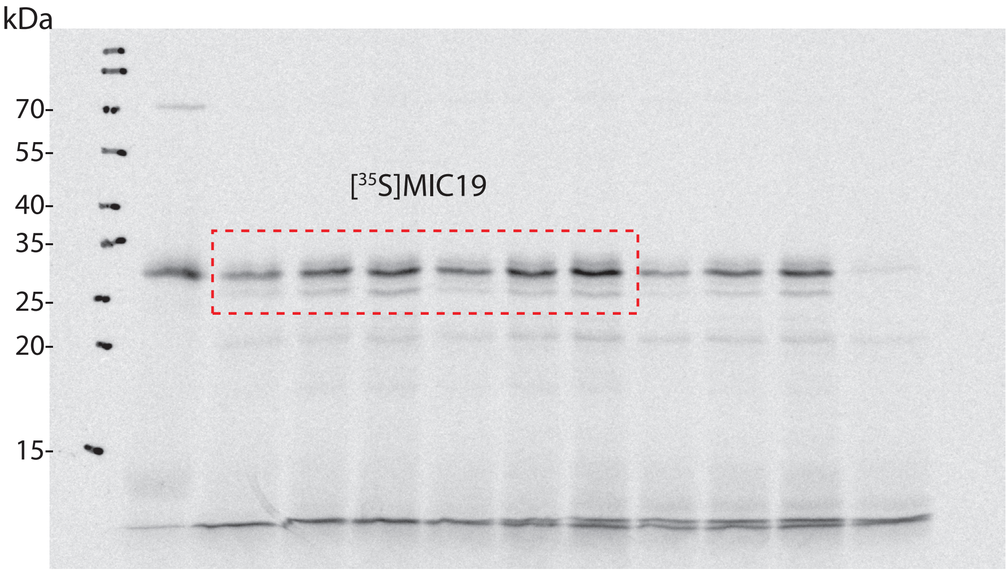

Figure 3f

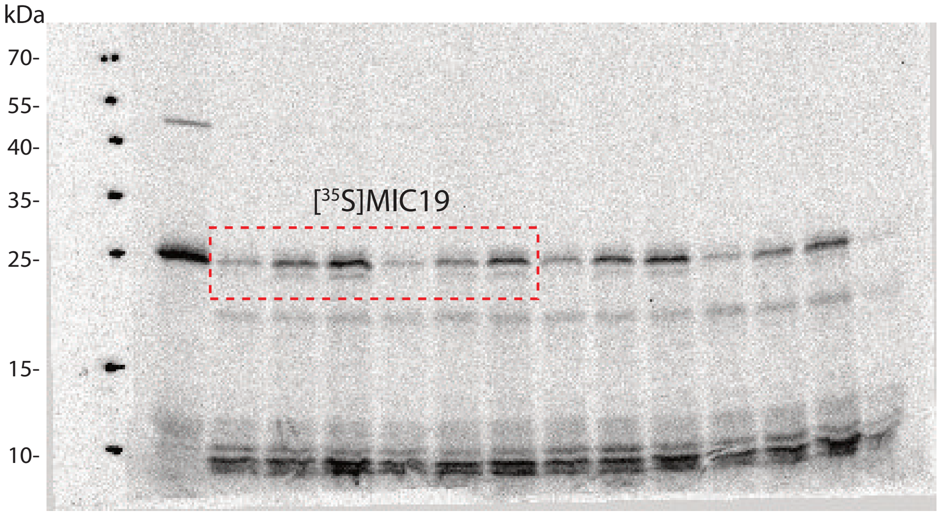

Figure 3g

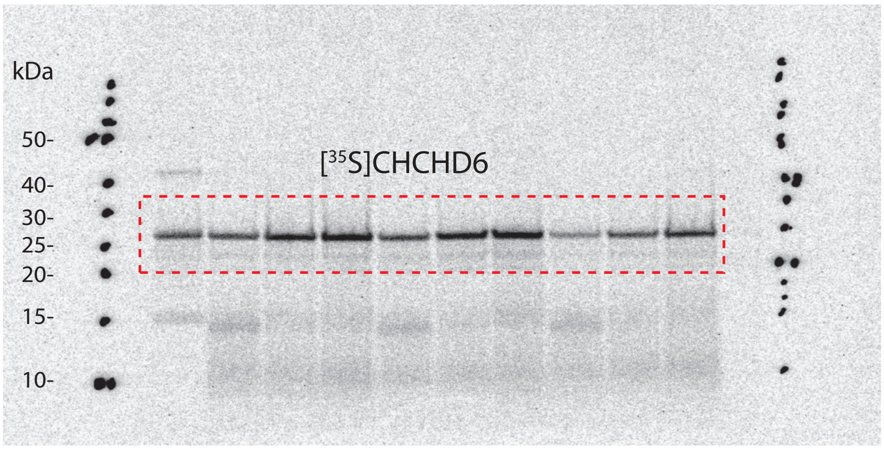

Figure 4b

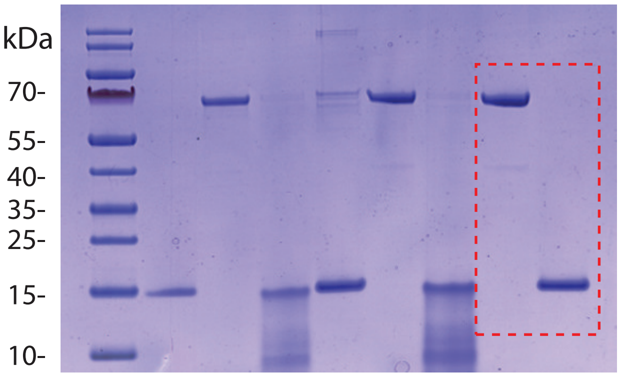

Figure 4c

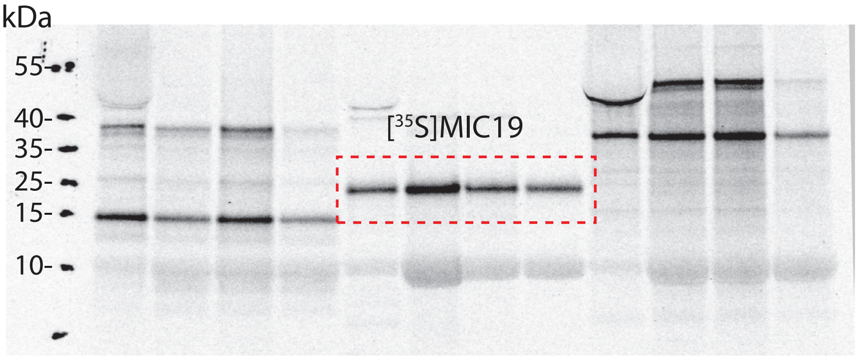

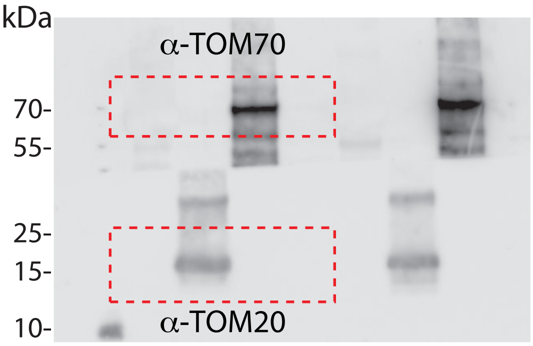

Figure 4d

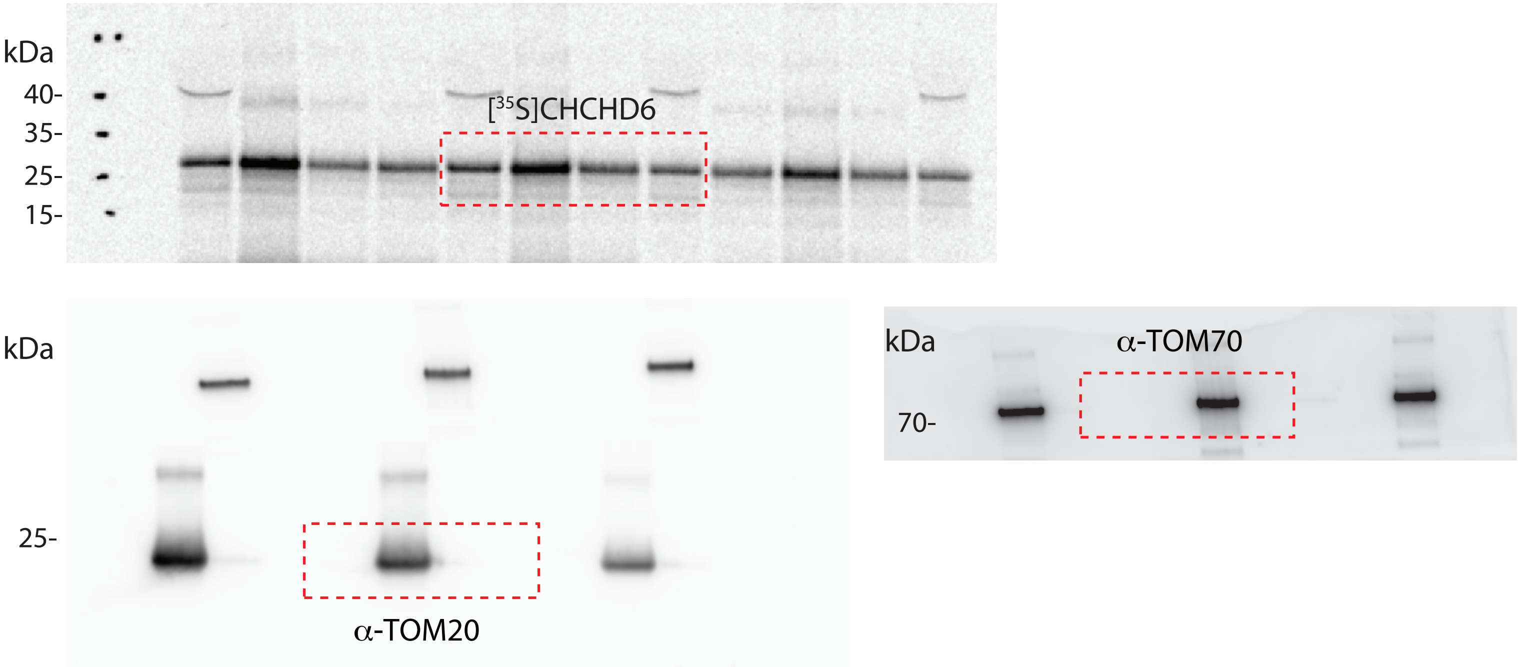

Figure 4e

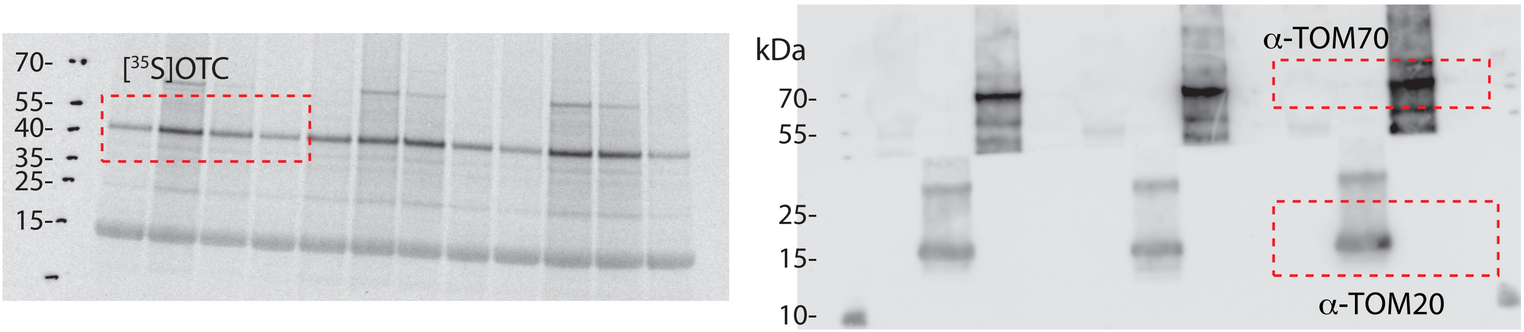

Figure 4f

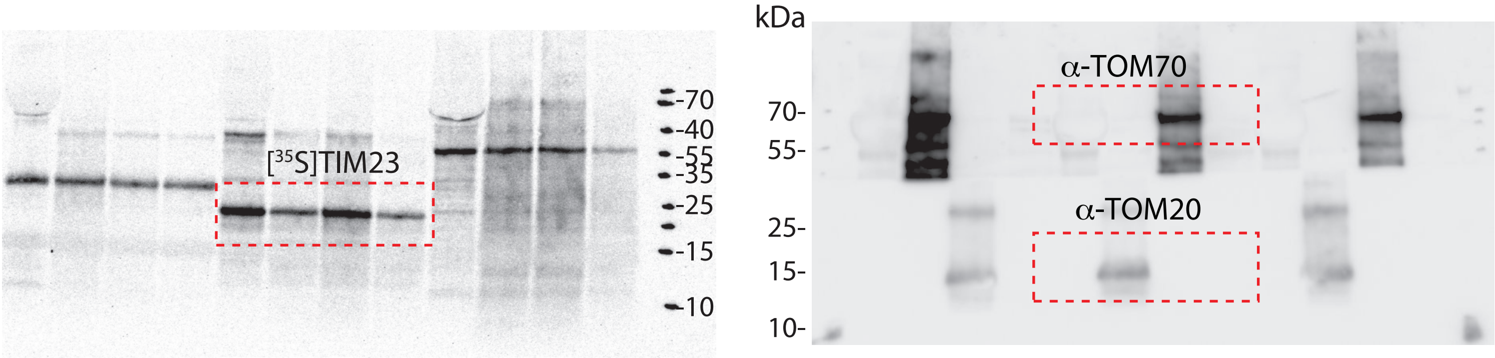

Figure 5a

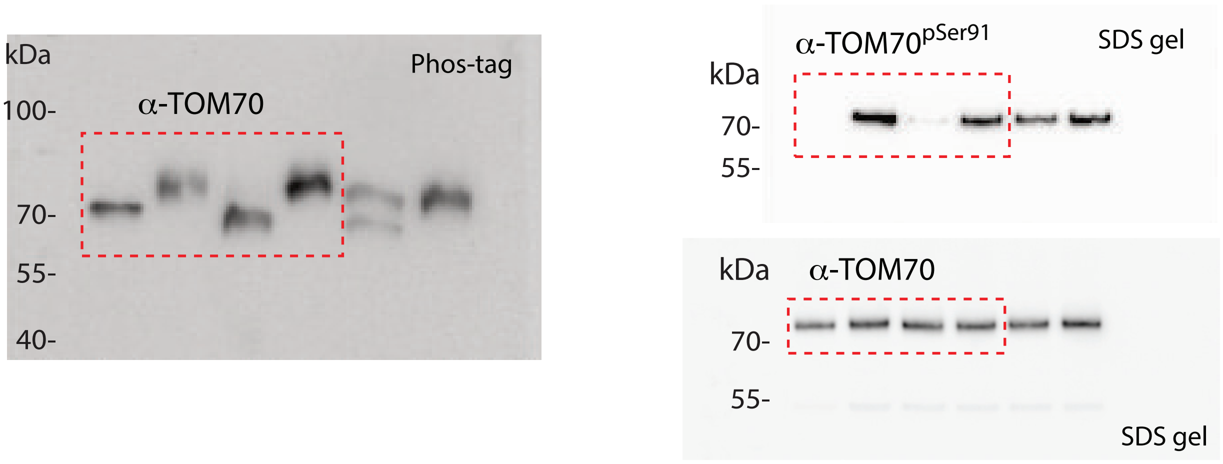

Figure 5b

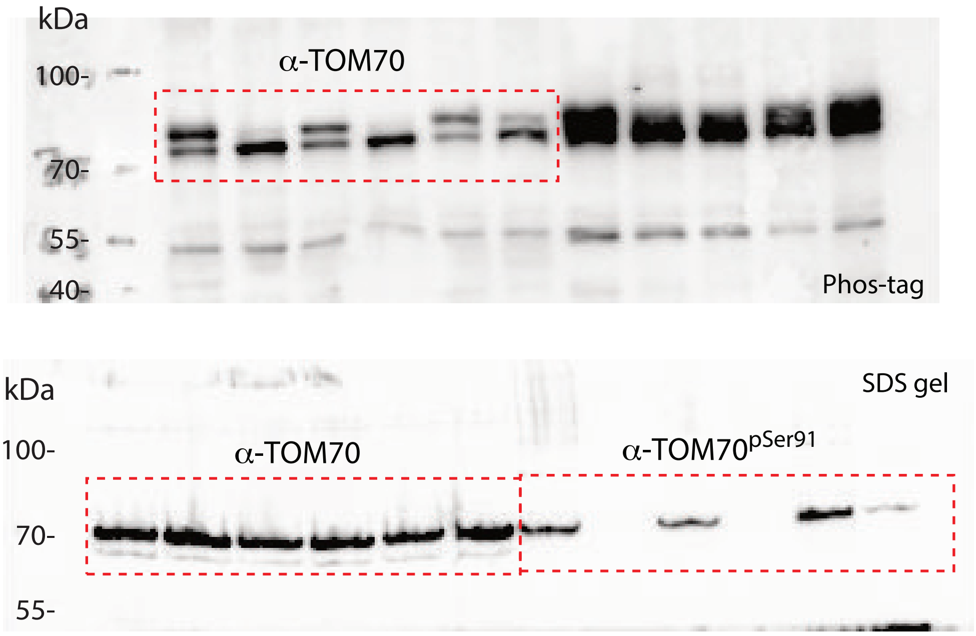

Figure 5c

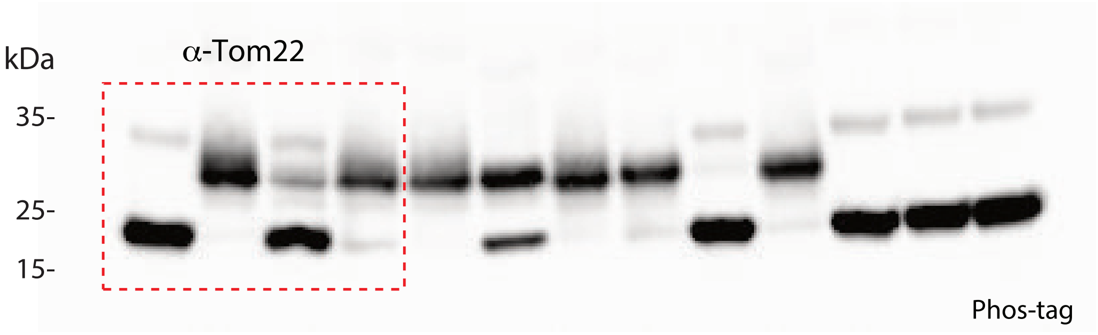

Figure 5e

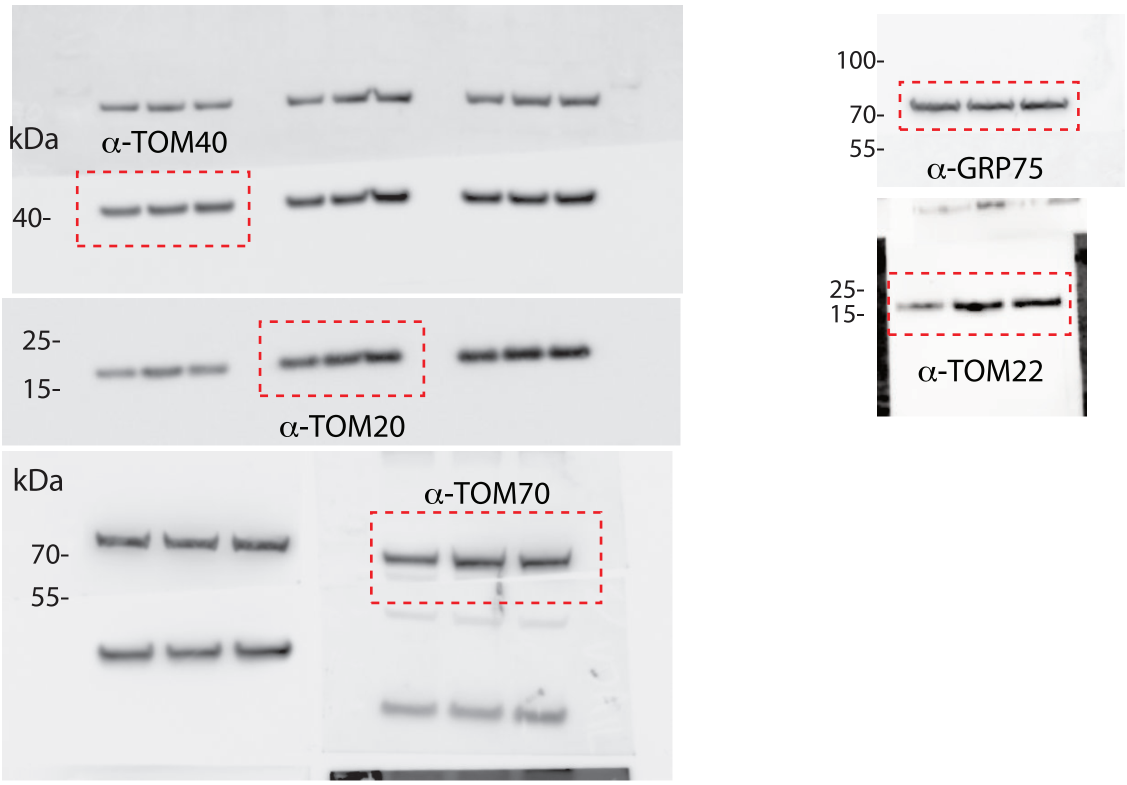

Figure 5g

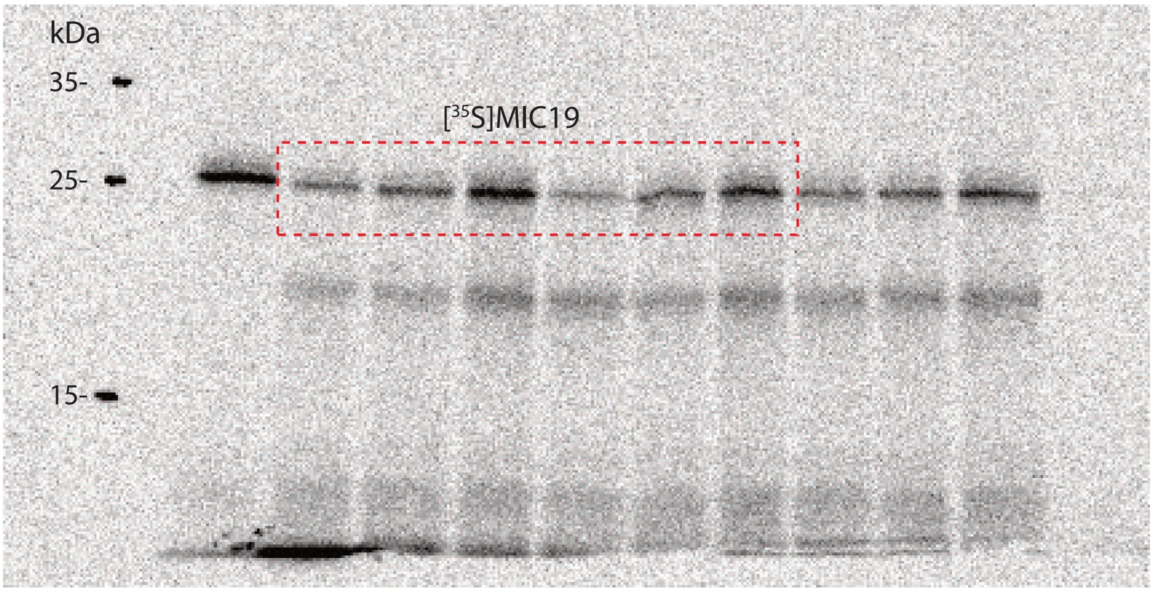

Figure 6a

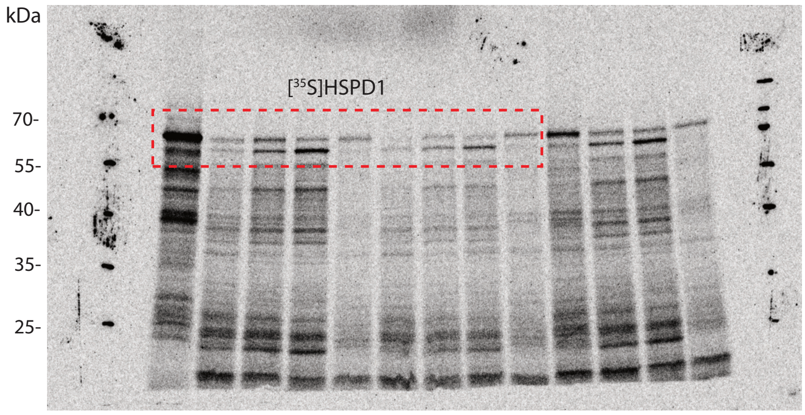

Figure 6b

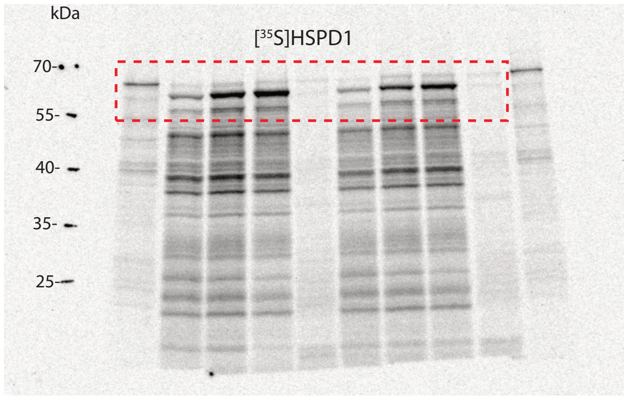

Figure S1a

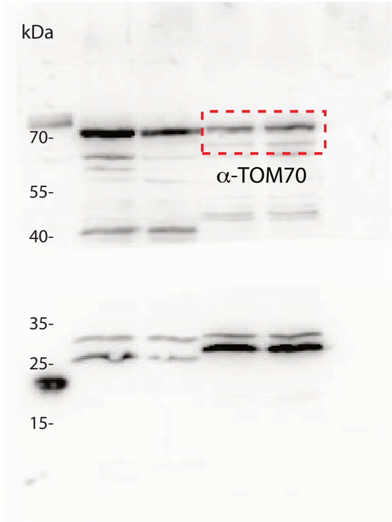

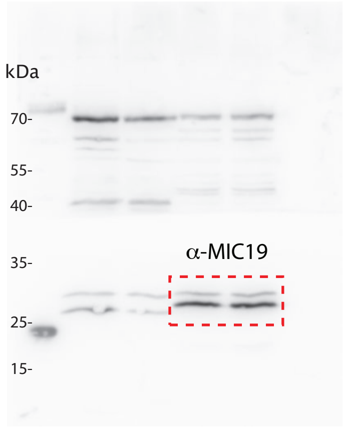

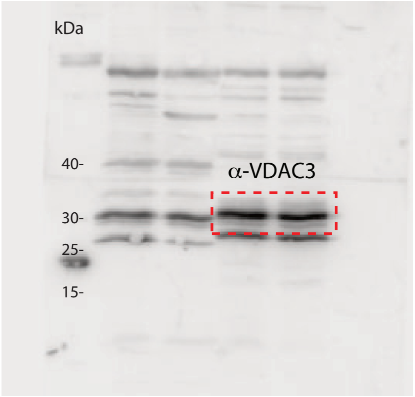

Figure S1b

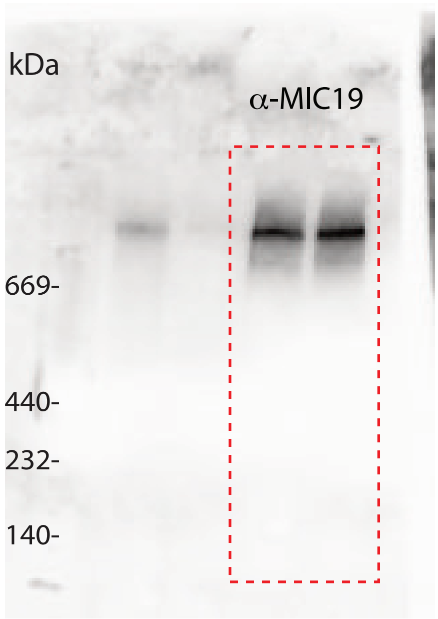

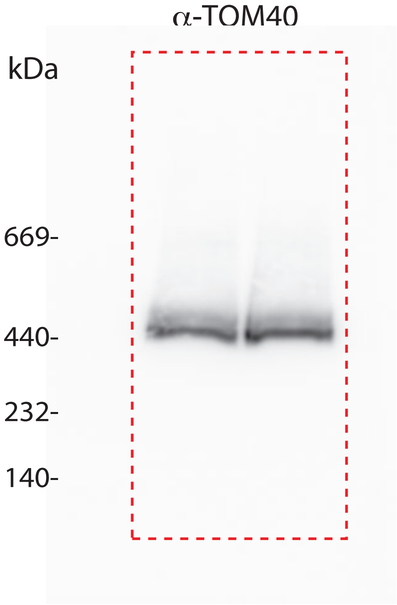

Figure S1c

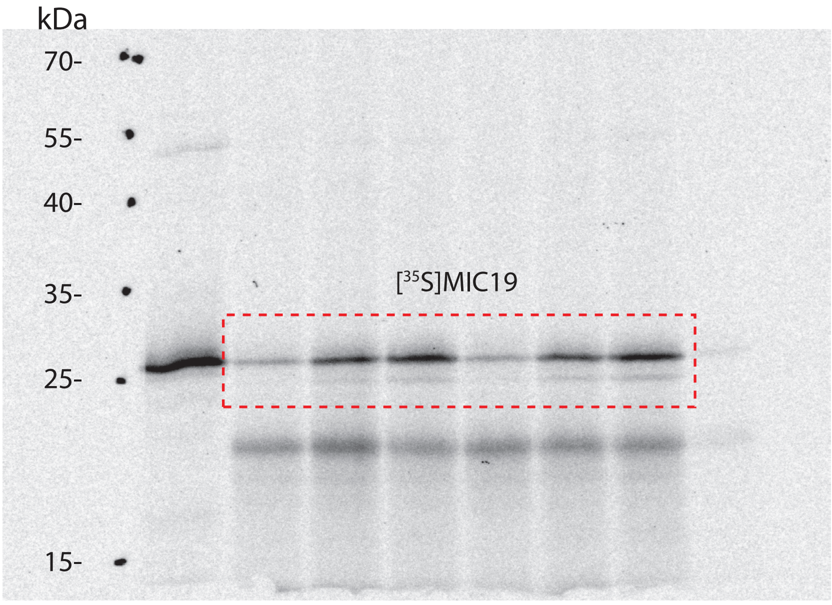

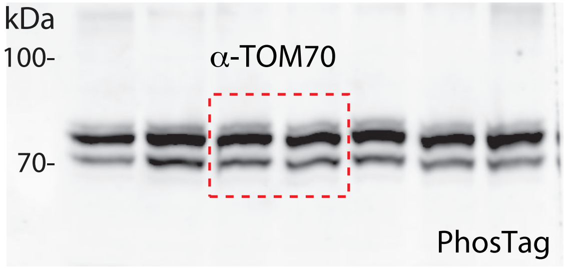

Figure S1d

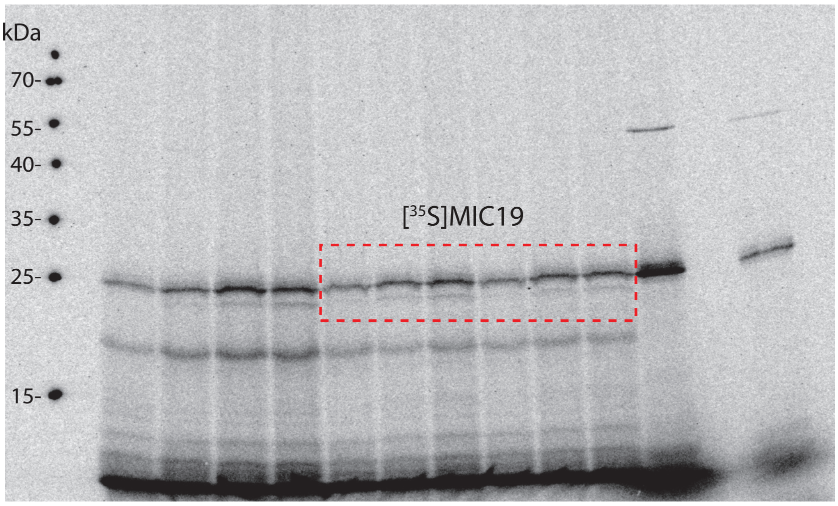

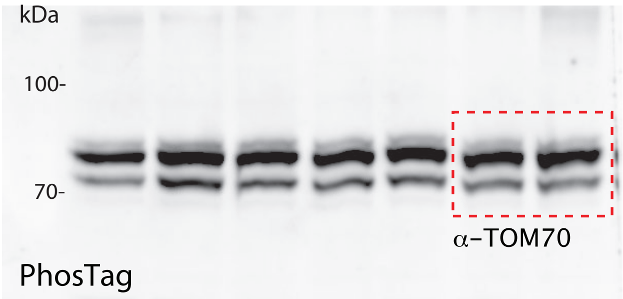

Figure S2a

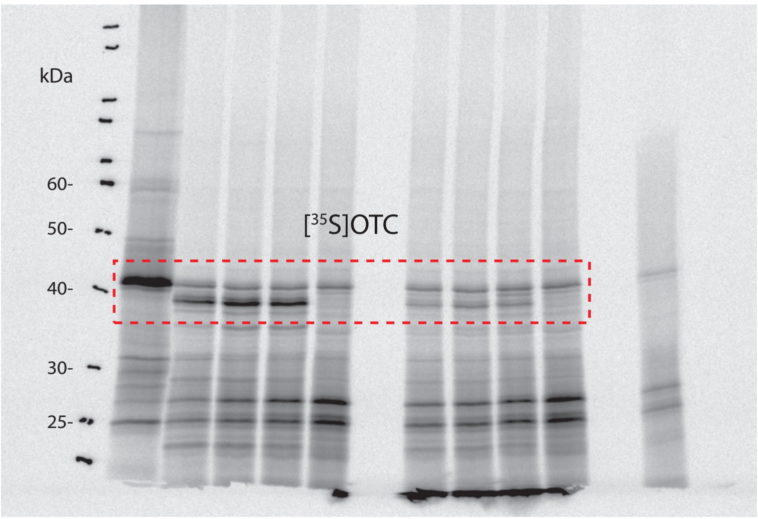

Figure S2b

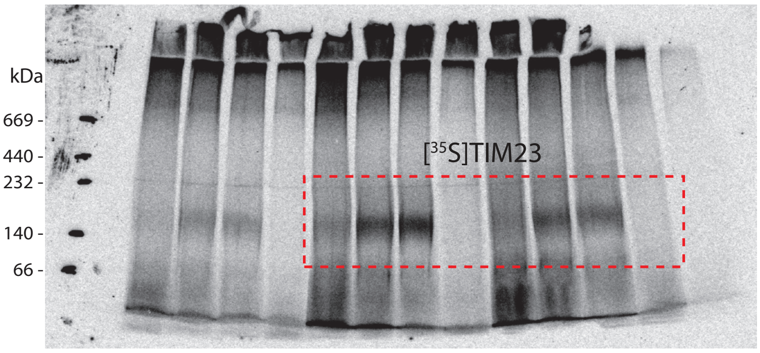

Figure S2c

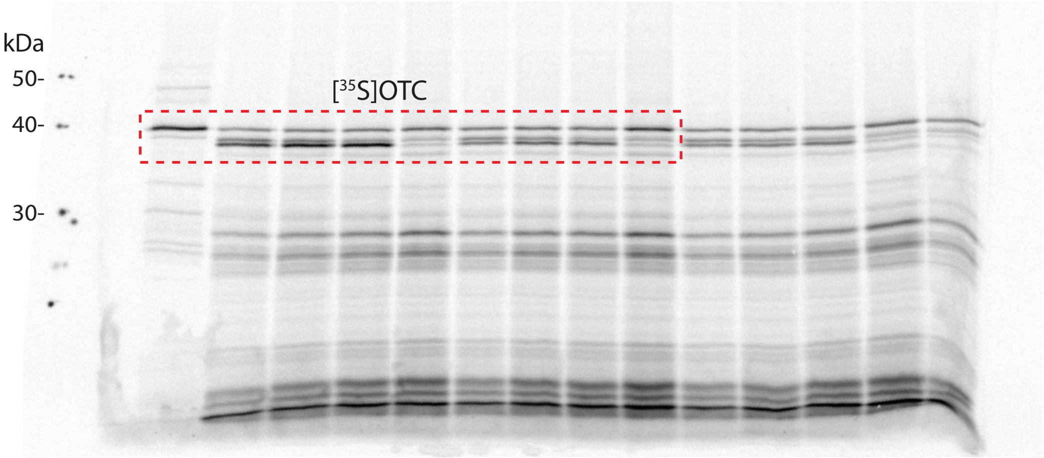

Figure S2d

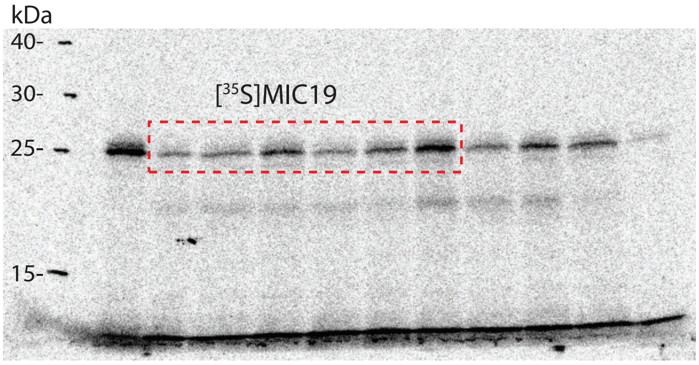

Figure S2e

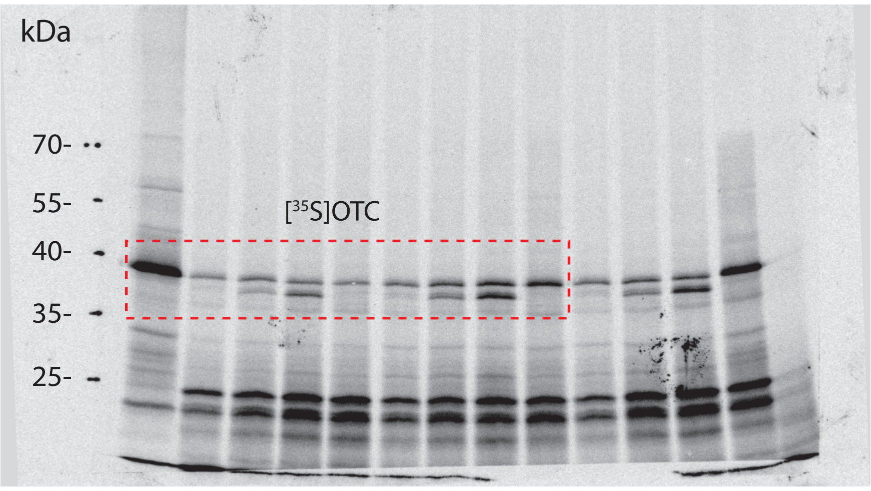

Figure S2f

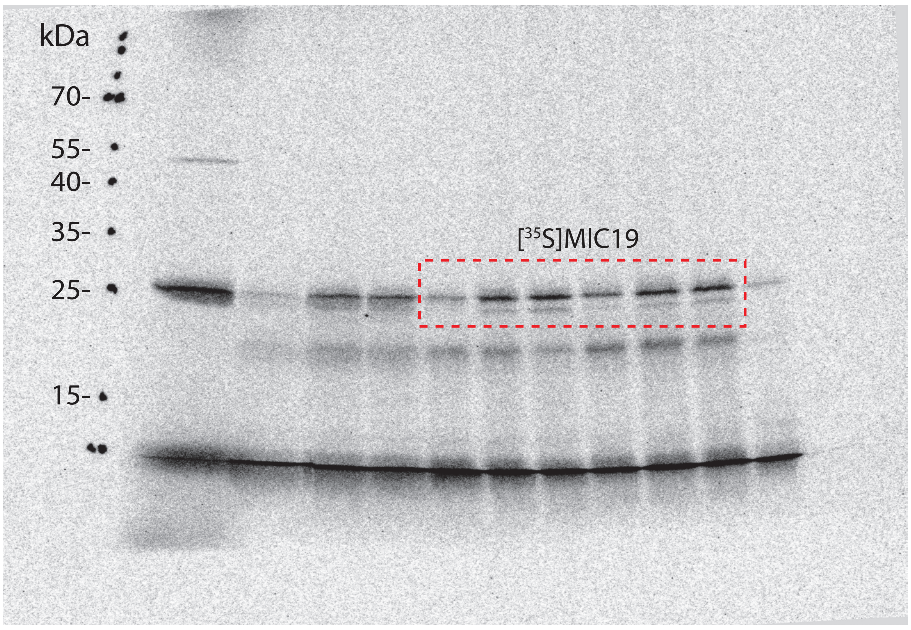

Figure S2g

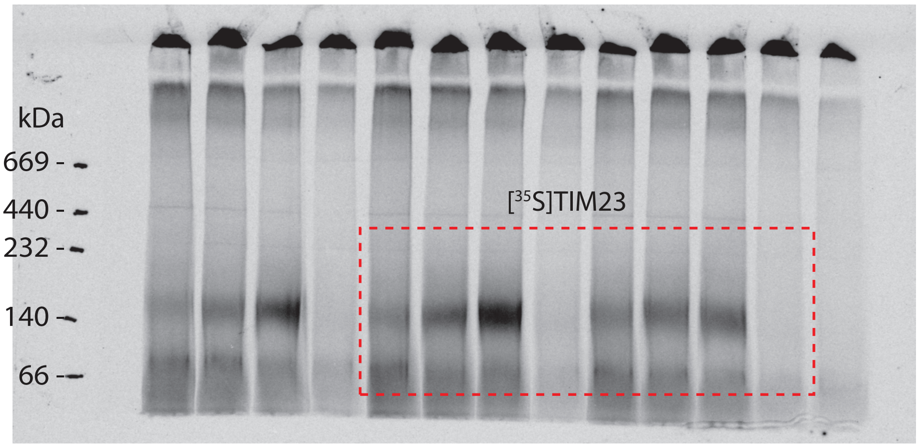

Supplement: Supplementary file 4 — Source data [file 41467_2024_49611_MOESM4_ESM.zip › Source data file_Marada et al_finalRevised.pdf]
